# Advances in Crack-Based Strain Sensors on Stretchable Polymeric Substrates: Crack Mechanisms, Geometrical Factors, and Functional Structures

**DOI:** 10.3390/polym17070941

**Published:** 2025-03-30

**Authors:** Chiwon Song, Haran Lee, Chan Park, Byeongjun Lee, Jungmin Kim, Cheoljeong Park, Chi Hung Lai, Seong J. Cho

**Affiliations:** Department of Mechanical Engineering, Chungnam National University, Daejeon 34134, Republic of Korea; song7367@o.cnu.ac.kr (C.S.); lhr0823@o.cnu.ac.kr (H.L.); cksdl4608@naver.com (C.P.); leebyeongjun@o.cnu.ac.kr (B.L.); kjm0689@o.cnu.ac.kr (J.K.); pffiro@o.cnu.ac.kr (C.P.); 202451063@o.cnu.ac.kr (C.H.L.)

**Keywords:** crack-based strain sensor, crack mechanism, geometrical factor, functional structure, numerical analysis, 3D crack

## Abstract

This review focuses on deepening the structural understanding of crack-based strain sensors (CBSS) on stretchable and flexible polymeric substrates and promoting sensor performance optimization. CBSS are cutting-edge devices that purposely incorporate cracks into their functional elements, thereby achieving high sensitivity, wide working ranges, and rapid response times. To optimize the performance of CBSS, systematic research on the structural characteristics of cracks is essential. This review comprehensively analyzes the key factors determining CBSS performance such as the crack mechanism, geometrical factors, and functional structures and proposes optimization strategies grounded in these insights. In addition, we explore the potential of numerical analysis and machine learning to offer novel perspectives for sensor optimization. Following this, we introduce various applications of CBSS. Finally, we discuss the current challenges and future prospects in CBSS research, providing a roadmap for next-generation technologies.

## 1. Introduction

As interest in the fields of wearable devices [1,2,3,4], healthcare monitoring [5,6,7], human–machine interface [8,9,10], and structural health monitoring [11,12] increases, interest in strain sensors based on flexible and stretchable polymer substrates is also increasing [13,14,15]. Among flexible and stretchable strain sensors, resistive strain sensors are particularly noteworthy due to their high sensitivity, good dynamic performance, and fast response time compared to capacitive or optical strain sensors [14]. In addition, resistive strain sensors have the advantage of not requiring complex interfaces for measurement and can be manufactured using simple processes [15].

Crack-based strain sensors (CBSS) have emerged as an innovative resistive sensor that achieves superior performance in sensitivity and working range by utilizing intentionally formed and propagated cracks [16,17,18,19,20,21,22,23,24]. By reframing cracks traditionally regarded as defects into functional elements, CBSS have driven significant advancements in wearable electronics [25,26,27,28,29], healthcare monitoring [30,31,32,33,34], human–machine interfaces [35,36,37,38,39], and structural health monitoring [40,41,42,43]. Notably, this crack-based approach enables ultra-high gauge factors (10^3^ to 10^8^ in some reports), strain detection exceeding 100% in certain designs, and rapid response times [44,45,46].

Despite these remarkable accomplishments, critical challenges remain, necessitating a more systematic exploration of CBSS. A wide array of crack configurations from straight and network cracks to more advanced kirigami and meta crack designs entails diverse trade-offs between sensitivity and working range [36,47,48,49]. Straight cracks and meta cracks contribute to high-sensitivity CBSS due to the rapid increase in resistance caused by the abrupt destruction of the conductive path, but this comes at the cost of a limited working range [47,49,50,51,52,53]. In contrast, network cracks and kirigami cracks provide a wider working range by maintaining conductive pathways through conductive bridges or stress distribution via out-of-plane deflection, yet their slower resistance increases leads to lower sensitivity [36,47,48,54,55,56,57,58,59]. Additionally, geometric parameters (e.g., asperity height, crack depth, and crack density) exhibit complex interdependencies, making precise control difficult [60,61]. Functional structures such as stress concentration patterns, wrinkle interfaces, and porous architecture further show promise for performance enhancements, yet their large-scale, high-precision manufacturing remains a significant hurdle [62,63,64].

While existing reviews often focus on individual elements such as a specific crack type or mechanical characteristic, they seldom provide an integrated view that ties together mechanisms, geometrical factors, and functional structures [65,66,67,68]. This gap hinders a broader understanding of how these features collectively influence sensor behavior and application potential. Furthermore, mathematical and physical theoretical models [69], as well as data-driven predictive modeling methods such as machine learning [70,71] and knowledge graphs [72] have shown promise in guiding the design and performance prediction of CBSS. These approaches help reduce the trial-and-error process in numerous experiments by considering the complex interrelationships of various crack-related elements. However, they have not yet received sufficient attention.

**Figure 1:**
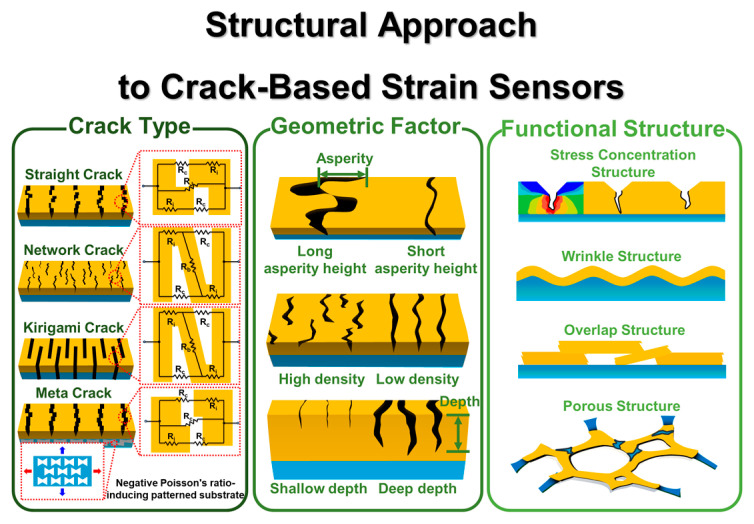
Schematic of structural approach to crack-based strain sensors: Crack Type, Geometrical factor and Functional Structure.

Therefore, this review seeks to address these gaps by presenting a comprehensive perspective on the interplay among crack mechanisms, geometrical factors, and functional structures in CBSS (Figure 1). We particularly highlight the roles of emerging computational tools, which can reduce experimental trial-and-error and expedite the development of highly performant and scalable CBSS. By systematically examining existing challenges and identifying new avenues for innovation, this review aims to offer researchers and engineers a roadmap to advance the field of CBSS, with potential implications in next-generation physiology and motion monitoring, human–machine interface, and structural health monitoring applications.

## 2. Mechanisms of Crack-Based Strain Sensors

**Figure 2:**
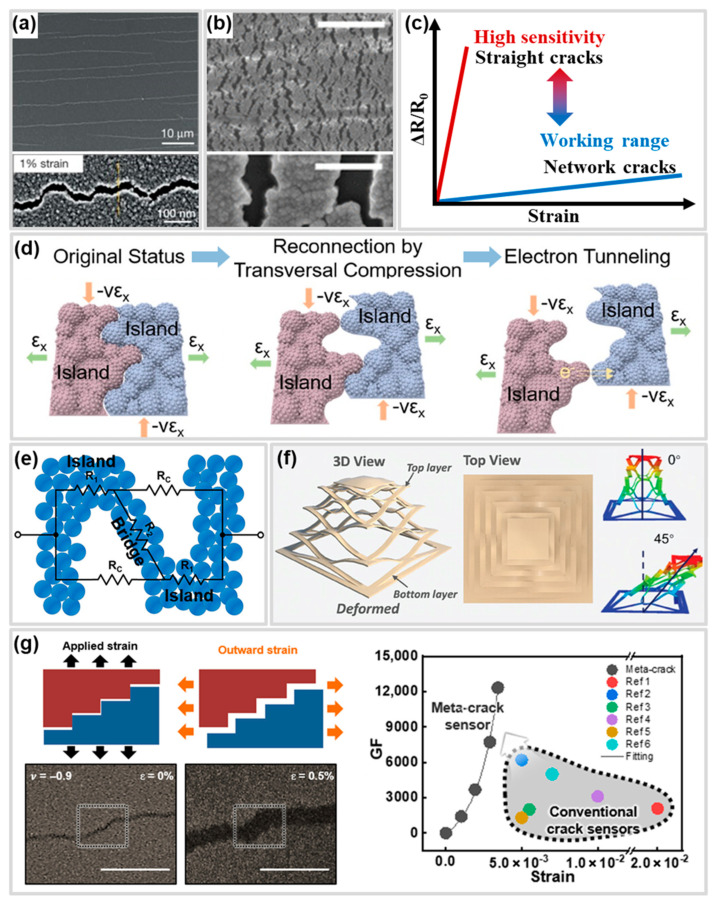
(**a**) SEM image of a CBSS with a straight crack shape [23]; (**b**) SEM image of a CBSS with a network crack shape [61]; (**c**) Sensitivity and working range of CBSS according to crack shape [28]; (**d**) Disconnection-reconnection process and tunneling effect [74]; (**e**) Island-bridge structure [61]; (**f**) Kirigami pattern and force distribution of the crack [75]; (**g**) Mechanisms and performance graph of meta crack [49].

Types of cracks are the primary factors that must be controlled to influence the performance of CBSS [47,73]. The performance is characterized by sensitivity and working range. Crack types generated in the conductive layer can be categorized into a straight crack shape (Figure 2a) and network crack shape (Figure 2b). These controlled crack types enable the attainment of desired performance of CBSS [28,66]. Generally, straight crack shapes are applied to CBSS for high sensitivity, as they result in the relatively rapid destruction of conduction paths in the conductive layer [23,74]. In contrast, the network crack shape is applied to CBSS with a wide working range due to the relatively slow conduction path destruction, which also involves intermittent tunneling effects as the crack asperities separate and reconnect (Figure 2c) [61]. In addition, the kirigami cracks [36,75] and meta cracks [49], unlike conventional crack types, are briefly introduced in the Advanced Crack Section. The kirigami cracks (Figure 2f) and meta cracks (Figure 2g) propose a novel crack opening mechanism using metamaterials to overcome the limitations of conventional crack opening mechanisms. These crack types utilize the tunneling effect during their initial deformation phases, which enhances their response time and sensitivity under small strains. These crack types and their performance, such as sensitivity, working range, and response time, are summarized in Table 1. In this section, we discuss the mechanisms and influence of various crack types, including the recently reported kirigami and meta crack, on the performance of CBSS. Furthermore, we introduce the latest studies, providing insights into their potential advancements in the field.

**Table 1:** Summary of the performance of CBSS with crack type.

Crack Type	Conductive Layer Material	GF_max_	Working Range (%)	Repeatability (Cycle)	Response Time (ms)	Ref.
Straight	Pt	2000	2	5000	100	[23]
	MWCNT	593.3	5	10,000	50	[76]
	AgNW	874.1	160	500	N/A	[77]
	* GNWs	8.6 × 10^4^	4	800	N/A	[50]
	Ag/AgNPs	1870	1.2	2000	0.252	[51]
	Ti/Au	9327	10	3000	72	[52]
	Au	15,000	18	12,000	11	[53]
Network	Pt	30	150	100	30	[61]
	Pt/AgNW/** DS	493.2	75	1000	205	[78]
	CNTs	83,982.8	300	10,000	70	[79]
	*** MXene-AgNW	224	60	6000	30	[80]
	AgNPs	>10	55	8000	16.3	[81]
	Au/Ti	113.7	120	1000	86.4	[47]
	CNTs	87	100	1500	65	[82]
	Ti_3_C_2_T*_x_* MXene	45	50	1000	N/A	[54]
	CNTs/CB	1516	500	1000	80	[83]
	CNT ink	8.9 × 10^4^	630	10,000	120	[84]
	AgNWs/CNTs	244.3	380	1000	80	[55]
	MXene/CNT	8089.7	450	10,000	40	[85]
	**** LSG	2692	120	11,000	N/A	[56]
	AgNWs/Au	6.1	350	1000	N/A	[57]
Kirigami	MXene	29.1	5000	1000	17	[36]
	Conductive hydrogel	26.9	25	N/A	N/A	[58]
	AuNP-MWCNT	220	180	100	10,000	[48]
	CB/MWCNT-TPU	5705.53	150	6000	220	[86]
	Pt	1220.71	2	5000	1700	[87]
	LIG	1.6 × 10^3^	30	1000	220	[88]
	Conductive hydrogel	80	215	10,000	85	[59]
Meta	Mo/MoO_3_/PBAT/PLA	12,331	0.34	6400	N/A	[49]

* GNWs: Graphene nanowalls. ** DS: Dragon skin. *** MXene: New intriguing family of 2D transition metal carbides, nitrides, and carbonitrides. **** LSG: Laser-scribed graphene.

### 2.1. Straight Cracks

The straight crack shape is defined as a crack shape that cuts across a conductive layer (Figure 2a) [23]. This crack shape is used to fabricate highly sensitive CBSS with high sensitivity. The sensitivity is expressed as the gauge factor (GF) which is defined as the rate of change of resistance per unit strain, formula: ((∆*R*/*R*_0_)/*ε*) [89]. When a certain strain is applied to a sensor composed of a conductive layer and a substrate, the formation of straight cracks can result from poor adhesion between the conductive layer and the substrate [90] or stress concentration [91] which localizes deformation within the conductive layer. This phenomenon leads to the rapid disruption of the conduction path and a significant increase in the total resistance of the conductive layer, even under small strains. The main mechanism of resistance change in a CBSS with a straight crack shape is the disconnection-reconnection process that occurs due to the separation and connection of micro-nano size crack asperities and the tunneling effect. When a strain is applied to a CBSS, cracks with asperities at their edges develop in the conductive layer. As the strain increases, the crack widens, increasing the distance between the separated conductive layers and disrupting the conductive path. However, due to Poisson effect, contact between crack asperities with nanoscale heights occurs, reestablishing the conduction pathway. This disconnection-reconnection process is repeated until the crack gap exceeds the crack asperity height, resulting in complete destruction of the conductive path (Figure 2d) [74].

Equations (1) and (2) show the normalized conductance of the conductive layer and relative resistance change in straight crack, respectively. These equations show the relationship between strain and Poisson’s ratio, and these equations are based on the disconnection-reconnection mechanism [23,61].

(1) $$S=\frac{1}{2}(1-\mathrm{erf}\left(\frac{ln(\varepsilon /\varepsilon_{0})}{\mu }\right))$$

(2) $$\frac{∆R}{R_{0}}=2{\left(1-erf(\frac{In(\varepsilon /\varepsilon_{0})}{\mu })\right)}^{-1}-1\;$$

*S* is the normalized conductance, erf(x) is the error function, *µ* is the Poisson’s ratio, *ε* is the strain, *ε*₀ is the initial strain, *R* is resistance, Δ*R* is *R* − *R*_0_.

The tunneling effect is the phenomenon of electrons passing through an insulating material where they are not normally allowed to move [92,93,94]. In CBSS, even when crack propagation disrupts the conduction paths formed by the contact of adjacent crack edges, electrons can still be transported across the crack via the tunneling effect, provided that the crack gap remains sufficiently small (Figure 2e) [61]. The tunneling resistance from the extremely small gap between conductive layers can be explained based on Simmons’ theory [95]:

(3) $$R_{tunnel}=\frac{V}{AJ}=\frac{h^{2}d}{Ae^{2}\sqrt{2m\lambda }}\exp\left(\frac{4\pi d}{h}\sqrt{2m\lambda }\right)$$

In the CBSS, *V*, *A*, and *J* represent the potential difference, the cross-sectional area of the tunneling junction, and the tunnel current density, respectively. *h*, *d*, *e*, λ, and *m* also represent Planck’s constant, the distance between adjacent conductive materials, the charge of an electron, the height of the nonconductive material’s energy barrier, and the mass of an electron, respectively.

In other words, within the strain range where the tunneling effect is dominant, it can contribute to the high sensitivity by causing a rapid increase in resistance [74,96]. Because the disconnection-reconnection process is generated by the disconnection and reconnection of micro-nanoscale crack asperities, and the tunneling effect also occurs at the nanoscale distance between adjacent conductive layers, these mechanisms act as crucial resistance change mechanisms in straight crack shape CBSS that have high sensitivity. As described above, the straight crack disconnection-reconnection mechanism and tunneling effect occur under very small strains. In addition, the straight crack can completely separate the conduction path between the conductive layers even at small strains (normally within 5%) [78], which is advantageous for designing high-sensitivity CBSS [23,50,51,52,53,74,76,77]. For example, Kang et al. [23] developed a CBSS with high sensitivity (GF 2000 at 0–2% strain) via a straight crack using platinum (Pt) on polyurethane acrylate (PUA). This crack sensor showed about 450-fold higher resistance variation at 0.5% strain compared to a non-crack Pt film sample. Also, Le et al. [74] reported that graphene oxide (rGO)/polydimethylsiloxane (PDMS) composite film with straight cracks formed by bending, exhibited high sensitivity due to disconnect-reconnect mechanisms and tunneling effects. They achieved the GF of 495 at strains of 0–0.55% through edge’s crack disconnect-reconnect mechanisms and a GF of 8699 at 0.8–1% strains due to tunneling effect.

### 2.2. Network Cracks

The network crack shape features an island-bridge structure, consisting of separated conductive islands where the conduction path is interrupted by microcracks, and conductive bridges that connect these separated islands (Figure 2e) [61,79,82,97].

The island-bridge structure is fabricated by delaying the strain localization of the conductive layer due to sufficient adhesion between substrate and conductive layer under deformation of the substrate [90]. The island-bridge structure of a network crack enables the conductive path to be maintained through the conductive bridge, even when the tunneling effect and disconnection-reconnection process fail to sustain the conductive path due to the gap between the separated conductive materials (conductive islands). The conductive bridge acts as a junction within the conductive path, facilitating electron transfer between the separated conductive islands. Because of this, CBSS with network crack shapes can generally have a wider working range than CBSS with straight cracks.

Jeon et al. [61] analyzed the network crack shapes with island-bridge structures using Pt/Polyurethane (PU) sensors. When the Pt/PU CBSS were subjected to tensile strain, the generation and growth of cracks in the Pt layer resulted in the formation of an island-bridge structure that contributes to wide working range of 150%, comprising separated conductive islands and conductive bridges connecting them (Figure 2e). The resistance of the network crack shape with the island-bridge structure generated in the Pt layer can be formulated as follows [61]:

(4) $$R=\frac{R_{1}R_{2}+2R_{1}R_{C}+R_{C}R_{2}}{R_{1}+2R_{2}+R_{C}}$$

In the above formula, *R*_1_ represents adjacent conductive islands, *R*_2_ is the resistance of the bridge that connects adjacent conductive islands, and *R_C_* is the resistance of cracks. If the electrical conductivity of substrate is very low, *R_C_* is assumed as an electrical insulator that has infinite electrical resistance, so Formula (2) can be simplified as follows:

(5) $$R=\frac{R_{1}R_{2}/R_{C}+2R_{1}R_{C}/R_{C}+R_{C}R_{2}/R_{C}}{R_{1}/R_{C}+2R_{2}/R_{C}+1}={2R}_{1}+R_{2}$$

Recently, attempts have been made to further enhance the working range of CBSS by forming additional conductive bridges in the crack gaps of the island-bridge structure using nanomaterials [55,79,82,83,84,85]. For example, the carbon nanotube (CNT) conductive layer with the island-bridge structure enabled electron transfer through the bridges, even when separated by cracks, demonstrating a wide working range [82]. Zhou et al. [79] reported spray-coating CNT ink onto a TPU fibrous mat, enabling CNTs to extend at the crack edges during crack formation and propagation, forming connections between islands. This design achieved a wide working range of 300% and high GF of 83,982.8 simultaneously.

### 2.3. Advanced Cracks

The Advanced Crack Section briefly introduces kirigami cracks, which are explained by crack propagation mechanisms like those of straight and network patterns, and meta cracks, which propose a novel crack opening mechanism utilizing metamaterials to address the limitations of conventional crack opening mechanisms.

Kirigami cracks are formed through patterns created by cutting the substrate or conductive layer, enabling the structure to withstand various deformations such as tension, bending, and twisting (Figure 2f) [35,75,98]. These cracks utilize the distortion of the cut patterns induced by deformation as a mechanism for crack opening and propagation, thereby altering the conductive paths within the conductive layer. Wu et al. [98] developed a kirigami CBSS by applying mechanical cuts to a silver nanowire (AgNW)/PDMS composite, fabricating periodic cutting patterns. These patterns caused changes in the conductive path within the sensor as cracks opened and propagated, resulting in resistance change. They investigated slit depth, slit length, and slit pitch to improve the performance of sensors. These kirigami structures can simultaneously improve sensitivity and the working range of CBSS by optimizing the stress concentration areas of the patterns. Zhuo et al. [36] developed a kirigami crack pattern using 3D printing with hydrogel. The developed kirigami-inspired hydrogel sensor generated stress concentration at the intersections of the kirigami crack pattern under tension, with the edges bending. This provided a sensitivity of 29.1 in the strain range of 140–500%, representing an approximately 5.5-fold increase compared to hydrogel sensors without kirigami patterns. Furthermore, kirigami crack patterns can enhance durability. Meng et al. [99] fabricated a serpentine pattern with open cracks of controllable widths and densities on a conductive TPU film using laser cutting. The patterned film was then transferred onto a pre-stretched elastic TPU film, forming closed cracks. The cracks in the fabricated kirigami pattern result in a high GF of 495 and a low detection limit (0.01%) at 8.7% strain. Additionally, the conductive thin film maintained its conductive pathways even under strains exceeding 50%, beyond the working range. While the sensor exhibited a significant increase in resistance under twisting and tension, it showed minimal resistance change under out-of-plane deformations, such as bending. The previously discussed studies suggest that kirigami cracks can provide various performance advantages depending on the patterning method. Consequently, these kirigami CBSS have powerful potential usages where high sensitivity and broad working range are required; measuring mechanical stress over the working range, detecting out-of-plane deformation [71,100].

Meta cracks (Figure 2g) enable extremely high sensitivity, even within very small strain ranges, by preventing the reconnection of crack edges using a substrate with a negative Poisson’s ratio. Lee et al. [49] were the first to introduce meta cracks into CBSS by controlling the Poisson’s ratio of the substrate to a negative value, achieving a highly sensitive CBSS capable of detecting strains as small as 10^−5^. The sensor consisted of a molybdenum (Mo)/molybdenum trioxide (MoO_3_)/polybutylene adipate terephthalate (PBAT)/polylactic acid (PLA) structure, where an auxetic pattern applied to the PLA layer imparted a Poisson’s ratio of −0.9 to the PBAT/PLA substrate. This design facilitated biaxial stretching of cracks in the Mo layer and allowed the crack gaps to open instantly.

## 3. Effect of Geometrical Factors on Crack-Based Strain Sensors

**Figure 3:**
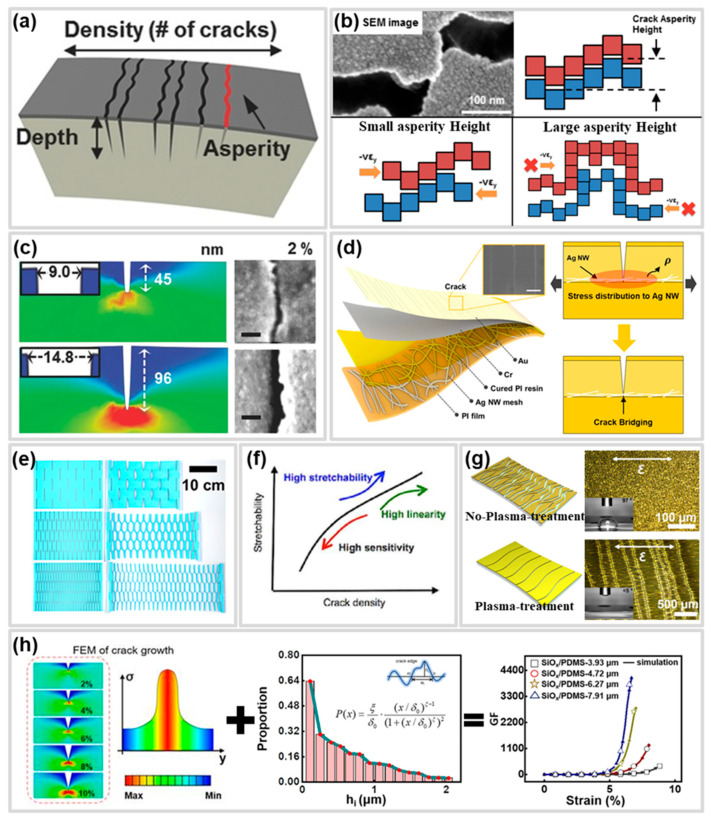
(**a**) Schematic of the geometrical factor of a crack in a CBSS [20]; (**b**) Contact of crack edge according to asperity height [23]; (**c**) Crack gap according to crack depth [20]; (**d**) Crack depth control with crack arresting layer [109]; (**e**) Crack growth model at various crack densities [61]; (**f**) Effect of density on performance in CBSS [110]; (**g**) Crack density control using surface modification [113]; (**h**) Performance prediction of CBSS by combining crack analysis with FEM simulation and mathematical theory model [116].

Geometric factors of cracks serve as an additional control mechanism that allows for precise tuning of sensor sensitivity and working range by adjusting specific characteristics of individual cracks, such as asperity, depth, and density (Figure 3a) [20,101]. Asperity is defined as the protrusion of a rough surface at the edge of a crack. Depth is defined as the height from the surface of the crack to the crack tip. Density is defined as the number of cracks per unit area or unit length. These geometrical factors significantly impact the performance of CBSS [67]; however, controlling these factors is essential yet challenging due to the limitations of experimental methods. Therefore, recent efforts have been made to optimize sensor performance through numerical analyses, such as computational models, finite element method (FEM) simulations, and machine learning [102]. In this section, we discuss the mechanisms and influence of various geometrical factors, including numerical analysis, on the performance of CBSS. Additionally, Table 2, unlike previous reviews, is a brief list of prior works that controlled geometrical factors and their corresponding sensor performance. Furthermore, we introduce the latest studies, providing insights into their potential advancements in the field.

**Table 2:** Summary of the performance of CBSS with crack geometric factor.

Geometric Factor	Crack Type	Conductive Layer Material	GF_max_	Working Range (%)	Repeatability (Cycle)	Response Time (ms)	Ref.
Asperity	Straight	Pt	2000	2	5000	100	[23]
	Straight	ITO	4000	2	5000	1	[103]
	Straight	PtNPs/Al_2_O_3_	2 × 10^4^	1	1200	100	[104]
	Straight	PtNPs/Al_2_O_3_	2.6 × 10^8^	7.2	1000	N/A	[44]
	Straight	PEDOT:PSS	280	8	2000	N/A	[105]
	Straight	Cu-Al	43,152	2	10	<100	[18]
	Network	MoO_3_/Mo	1355	2	10,000	N/A	[17]
Depth	Straight	Pt	16,000	2	10,000	N/A	[20]
	Straight	Au/Cr/MoO_3_	10,000	2	15,000	5	[106]
	Straight	Carbon paste	43.79	3.27	100,000	N/A	[107]
	N/A	CNT	43.8	200	1000	100	[108]
	Straight	Au/Cr/AgNWs	2000	44.4	200,000	0.2	[109]
Density	Network	Pt	30	150	N/A	30	[61]
	Straight	Au	5000	1	1000	N/A	[94]
	Straight	SWCNT	4.2 × 10^4^	153	5000	N/A	[110]
	Straight	CNTs	1020.2	100	10,000	N/A	[111]
	Network	MXene/CNT	89.72	100	10,000	353	[112]
	Straight	Au	10,000	120	5000	N/A	[113]
	Straight	Au	2448	59	3000	69	[114]
	Network	Ag	4570.6	50	1000	N/A	[21]
	Straight	Laser-induced graphene (LIG)	191.55	50	1500	70	[115]

### 3.1. Crack Asperity Height

Asperity, defined as the protrusion of a rough surface at the edge of a crack, is a crucial factor in determining the maintenance and destruction of the conduction path under strain, and subsequently, the sensitivity of CBSS [23,44,103,104]. When a CBSS is subjected to strain, cracks that have rough asperities form at the edge. Through a disconnection-reconnection mechanism, crack asperities partially come into contact and separate, maintaining partially active conductive paths. These partially active conductive paths are maintained until the crack gap exceeds the crack asperity height, and when the crack gap exceeds the crack asperity height due to larger strain, electrons cannot be transferred except through the tunneling effect, and finally a complete disconnection of the conduction paths occurs (Figure 3b) [23,104].

Therefore, adjusting the asperity height to a lower level can be utilized as a strategy to enhance the sensitivity of the CBSS by inducing a rapid change in the resistance of the conductive layer. The asperity height effect on the sensitivity of the CBSS can be clarified by formalization. Wu et al. [105] deduced the sensitivity through monitoring of morphological evolution with strain of Poly(3,4-ethylenedioxythiophene) polystyrene sulfonate (PEDOT:PSS)/fluorosilicone rubber (FSR) strain sensor in which disconnection-reconnection process acts as a sensing mechanism, the deduced formula takes total crack number (*N*), average asperity height ($\bar{h}$), initial length of sensor ($L_{0}$), and strain (ε) as variables and is formulated as follows.

(6) $$GF=\frac{1}{\frac{N\bar{h_{i}}}{L_{0}}-\varepsilon }$$

The effect of asperity on the performance of a CBSS through the disconnection-reconnection process can be illustrated by a comparison of the papers by two studies. Puyoo et al. [104] described that the average asperity height and standard deviation of the cracks generating in the Platinum nanoparticles (Pt Nanoparticles (NPs)) in Aluminum Oxide (Al_2_O_3_) thin film were lower than cracks in the Pt thin film of the CBSS by Kang et al. [23], thus enabling them to fabricate a CBSS with improved sensitivity. In addition, they also reported that a decrease in average crack height can reduce the strain point at which the GF is maximized.

Recently, methods have been reported to increase crack asperity height by adjusting the elemental composition and alloy thickness to induce large clusters or by controlling the deposition temperature to form larger grain sizes [17,18]. Controlling asperity height through adjustments in thickness and temperature helps reduce the probability of crack-edge reconnection, thereby improving sensor sensitivity.

### 3.2. Crack Depth

Crack depth, defined as the distance from the crack surface to its tip, plays a crucial role in enhancing the sensitivity of CBSS by inducing a wider crack gap at the same strain level as the depth increases (Figure 3c) [16,20,106,107,116,117]. To investigate the effect of crack depth on the sensitivity of sensors, Park et al. [20] mechanically bent the curved surface of a multi-layered sensor to create an initial crack and controlled the crack depth by applying additional tensile force. As the tensile force increased, the crack depth became deeper, resulting in a wider crack gap and, consequently, a higher GF of 16,000. There are various strategies for increasing crack depth such as thickness control of conductive layer, repeat cycling, and changing materials. Lee et al. [60] demonstrated that controlling crack depth by adjusting the thickness of the metal layer could optimize sensor sensitivity. By fixing the gold (Au) layer and varying the chromium (Cr) layer thickness, they found that thicker adhesion layers induced deeper cracks, leading to wider crack gaps and improved sensitivity. Kim et al. [106] reported two strategies, changing the substrate material and applying repeated cycles, for increasing crack depth in CBSS with an Au/Cr/MoO_3_ conductive layer. They achieved a 10-fold increase in sensitivity by replacing the PET substrate with Polyimide (PI) and further enhanced crack penetration depth and sensitivity through repeated tensile-compressive cycles. However, while inducing deeper cracks enhances sensitivity of CBSS, it can also lead to performance degradation or failure due to crack propagation during repeated use. To address this issue, Kim et al. [109] proposed inserting an AgNW mesh as a crack-arresting layer to control crack depth and suppress unwanted crack propagation (Figure 3d). This approach not only achieved a high GF of 2000 at 0.5% strain but also demonstrated exceptional durability, maintaining consistent sensitivity over 200,000 cycles.

### 3.3. Crack Density

Crack density, which is defined as the number of cracks per unit area or unit length [118], is a significant geometric factor influencing the sensitivity and working range of CBSS, alongside crack asperity and depth [61,94,110,119]. Jeon et al. [61] reported the effect of conductive layer thickness on the density of CBSS. They showed that the failure strain increases as the crack density increases since increased crack density has a high spatial capacity. As shown in Figure 3e, they showed this principle with the simple paper model and CBSS fabricated with thin Pt-deposition on PU membrane showing improvement of working range to 150% strain. Adjusting crack density is a critical geometrical factor in controlling both the working range and sensitivity of sensors (Figure 3f). Therefore, various methods have been employed to achieve these performances, including tuning the thickness of conductive layers [61,120], modifying substrate surfaces [111,112,113], and introducing stress-concentrating structures [110,114,121]. Mahmoud et al. [120] reported that the crack density can be controlled by controlling the thickness of the conductive layer to optimize the sensitivity and durability. In their experimental results, the conductive layer composed of Au/Cr showed a crack density that was inversely proportional to the thickness of Cr. In addition, as the crack density increased, the sensitivity and durability of the sensor tended to decrease. Li et al. [111] reported enhanced sensitivity in a CNT/Ecoflex sensor by employing ultraviolet (UV)/Ozone (O_3_) surface engineering to form low density straight cracks. These cracks were caused by a mismatch between Silicon Oxide (SiO_X_), formed on the Ecoflex surface during UV/O_3_ exposure, and the elastomer. As exposure time increased, the density of cracks in the CNT layer decreased, achieving a GF of 1020.2, which was significantly higher than the GF of 12 observed in sensors without UV/O_3_ exposure. Zhu et al. [113] demonstrated that plasma treatment could regulate the wettability of PDMS surfaces, thereby controlling the size of Au particles adhered to the surface. This allowed for modulation of crack density, which influenced both sensitivity and working range. P-PDMS sensors, subjected to plasma treatment, exhibited lower crack density and sensitivity due to larger particle sizes. In contrast, F-PDMS sensors, without plasma treatment, exhibited higher crack density and an extended working range because of smaller particle sizes (Figure 3g). Xin et al. [110] used laser engraving to create grooves on Single-Walled Carbon Nanotube (SWCNT) paper, inducing stress concentration and enabling precise control over crack density. This approach yielded diverse working ranges (60–153%) and a GF of 4.2 × 10^4^. However, direct processing of the conductive layer using lasers poses a risk of damaging the conductive layer. Wang et al. [114] used laser transmission pyrolysis (LTP) to fabricate microchannel arrays on PDMS substrates. This technique redistributed stress on the substrate, resulting in cracks in varying densities that simultaneously enhanced both sensitivity and working range. In the microchannel regions formed by LTP, the Au layer exhibited lower crack density, whereas the mesa regions displayed higher crack density. Consequently, sensors with mesa-like crack patterns achieved significantly higher sensitivity (GF ≈ 2448) and a broader working range of 59% suitable for detecting large deformations compared to sensors with flat surfaces.

### 3.4. Performance Control Through Numerical Analysis of Cracks Geometrical Factor

Numerical models and machine learning models for the geometrical factors of CBSS play a crucial role in optimizing their performance. Since the sensitivity and working range of CBSS are significantly influenced by the geometrical factors of cracks, a thorough process of optimization through experiments and analysis is essential for applying these sensors to various fields. However, such optimization processes demand substantial time, effort, and cost from researchers. Moreover, the interactions among the geometrical factors affecting sensor performance add further complexity to the optimization process. For these reasons, numerical models and machine learning models in CBSS research can provide new insights and serve as efficient analytical tools in terms of time and cost. In previous studies, researchers employed numerical approaches to analyze the effects of geometrical factors on CBSS. Wang et al. [115] optimized the balanced performance of sensitivity and working range in Laser-Induced Graphene (LIG)/Ecoflex sensors through a numerical model. The quality of LIG, used as the conductive layer, was optimized via Finite Element Analysis (FEA) by examining the thermal distribution caused by laser parameters during the laser scribing process. Furthermore, FEM simulations were utilized to explain experimental results by analyzing the geometrical changes, such as crack depth and gap opening, occurring during the tensile process, which demonstrated strong agreement with experimental data.

Guo et al. [107] investigated the changes in sensitivity of carbon paste-based strain sensors as crack density and depth increased, using Finite Element (FE) simulations, and confirmed that the results were consistent with experimental findings. Additionally, they observed through FE simulations that the stress intensity factor in the sensing layer decreased due to cracks and demonstrated a “crack arrest mechanism”, where crack growth halts as crack density increases. Based on these findings, they proposed a fatigue-based modulation strategy to enhance the long-term stability of sensors. Zhao et al. [117] developed an electrical–mechanical finite element model based on low-energy theory to predict the sensitivity and working range of crack-related information as a function. The proposed model was used to predict the effects of factors such as crack orientation/distribution, crack spacing, density, depth, and crack continuity on the sensing performance of CBSS. Similarly, Song et al. [122] combined information obtained from a three-dimensional mechanical model of a CNT-SiO_x_/PDMS sensor with electrical simulations using the probability distribution based cellular automata method (PDCA) algorithm. This approach enabled them to predict deviations in crack density and sensitivity due to strain, achieving prediction errors of 5.23% and 9.25%, respectively. Song et al. [116] also developed a predictive model for the crack geometrical factor and sensitivity of a PEDOT-SiO_x_/PDMS-based sensor by combining the mathematical relationship between crack depth, gap, and strain derived from theory of fracture mechanics with the mathematical model based on crack edge contact probability (Figure 3h). For a sensor with a 7.91 μm thick SiO_x_ layer, the prediction errors for crack depth, gap, and sensitivity were 3.75%, 5.74%, and 6.26%, respectively.

## 4. Functional Structures for Performance Improvement

Functional structures are structural modifications applied to the substrate or conductive layer to enhance the performance of CBSS. For instance, stress concentration structures focus stress on specific areas, accelerating the failure of the conductive layer due to cracks and thereby improving sensitivity [123]. Additionally, wrinkle, overlap, and porous structures delay the propagation of cracks in the conductive layer or prevent their rapid propagation, contributing to the improvement of the working range in CBSS [67,124]. Therefore, a deep understanding of functional structures and further research are essential for optimizing CBSS. For this reason, recent studies have actively explored hierarchical structures that combine two or more functional structures, along with numerical analyses and machine learning-based approaches to optimize functional structure designs. In this section, we aim to provide a comprehensive understanding of the effects of functional structures on CBSS by introducing the mechanisms and specific examples of each structure. This is summarized in Table 3, along with the materials. Additionally, we will briefly discuss numerical analysis and machine learning-based approaches for performance optimization.

**Table 3:** Summarized the performance of crack-based strain sensors with functional structure.

Functional Structure	GF_max_	Working Range (%)	Repeatability (Cycle)	Response Time (ms)	Material	Ref.
Stress concentration structure	2.0 × 10^6^	10	5000	100	Pt/Cr/PUA	[125]
	670	0.3	20,000	N/A	Au/Cr/Cu/PUA	[62]
	74	100	1000	N/A	SWCNT/PDMS	[126]
	4570.6	50	1000	N/A	Ag/cPDMS *	[21]
	1084.16	100	1000	489	Ecoflex-GNPs/PDMS	[127]
	18,000	0.65	7000	258	AgNPs/PDMS	[128]
	150,000	60	30,000	N/A	AgNWs/PDMS	[129]
	184.88	1.67	500	145	Pt/TPU	[130]
	1.42e8	0.085	1000	N/A	Au/Cr/PDMS	[131]
Wrinkle structure	167,665.6	300	10,000	90	Au/rGO/VHB/PDMS	[132]
	1.1 × 10^9^	125	3500	0.13	Ag/rGO/ PDMS	[45]
	1071	15	150	200	GNC/PDMS	[133]
	287.6	100	10,000	489	CNT-GO/ Elastic tape	[134]
	750	24	20,000	90	GNPs/TPU/ PDMS	[135]
	2000	100	1000	30	SWNT/GO/ PDMS	[136]
	2585	66.5	1000	N/A	Au/PDMS	[137]
	1344.1	200	10,000	88	CNTs/PU yarn	[32]
	2	600	10,000	153	CNT/SEBS rubber elastomer	[63]
	136,124.4	300	10,500	140	MXene/rGO	[138]
Overlap structure	30,000	3	1000	N/A	GNPs/PDMS	[119]
	2369.1	80	2500	600	MXene/PANIF/ Elastic rubber	[139]
	58.5	200	5000	42	CNTs/AgNPs/ Spandex fiber	[140]
	42,300	145	1000	N/A	CNT/Ecoflex	[141]
	150	25	1000	70	GnPs/PU	[142]
	1.0 × 10^7^	300	1000	158	Au/PDMS	[143]
	64.08	500	1000	56	CNT/Ecoflex	[144]
	4000	1	1000	N/A	AgNWs/GO/TPU	[145]
	150	82	5000	N/A	rGO/elastic tape	[146]
	475	14.5	500	N/A	AgNPs/rGO/PDMS	[147]
Porous structure	0.039	900	2000	60	CNTs/TPU	[64]
	97.1	320	9700	<200	MWCNTs/TPU	[148]
	3405	83	1000	60	MXene/CNC/TPU	[149]
	0.8	80	500	46	MXene/PU	[150]
	66,600	520	5000	~60	CNTs/TPU fiber	[151]
Hierarchical structure	57.2	530	1000	200	CNTs/TPU fiber	[152]
	5.9	150	1000	192	GO/rubber band	[153]
	2352.8	50	11,000	125.4	a-C **/PDMS	[154]
	2557.71	45	10,000	<130	Au/PDMS	[155]

* cPDMS; Carbon added PDMS. ** a-C; Amorphous carbon.

### 4.1. Stress Concentration Structure

**Figure 4:**
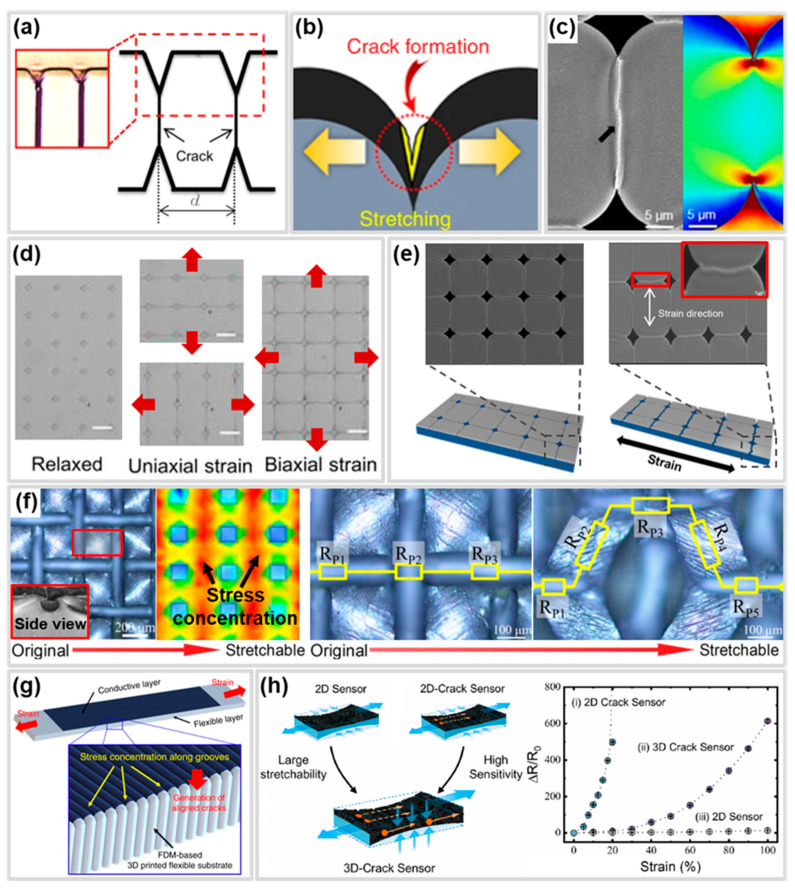
(**a**) Image and schematic of unidirectional crack control using notch structure in a double-layer system [156]; (**b**) Crack control using stress concentration in a CBSS [130]; (**c**) FEM analysis results of stress concentration structure in CBSS [125]; (**d**) Image of multidirectional crack control in a double-layer system using notch structure [158]; (**e**) Image of CBSS with controlled crack using notch pattern [125]; (**f**) FEM analysis image of groove structure and schematic of electrode path change according to crack formation [129]; (**g**) Schematic view of FDM-3D print based strain gauge [130]; (**h**) Schematic of 3D CBSS [127].

A stress concentration structure is a design that induces higher strain in a specific region by concentrating stress in that area. This structure includes various forms of protruded or recessed patterns, such as hall and line shapes (Figure 4a–c,f) [125,130,156]. Stress concentration structures can be utilized as a strategy to achieve high sensitivity in CBSS by controlling the location, direction, and rate of crack generation and propagation in the conductive layer of the CBSS, thereby facilitating a rapid increase in resistance [157]. Previous studies have reported that applying stress concentration structures, such as notches, in bilayer systems with different elastic moduli can effectively control cracks through localized stress concentration. Moreover, these structures enable the regulation of crack initiation and propagation regions and directions under various shapes and external force directions (Figure 4d) [158]. Choi et al. [125] reported the fabrication of a high sensitivity crack sensor by applying V-notch-shaped hole patterns to C-PUA using lithography, followed by Pt/Cr deposition. The hole patterns on the surface induce stress concentration around the holes, generating controlled straight cracks that rapidly separate the conductive layer (Figure 4c). These cracks propagate across the surface by connecting adjacent holes (Figure 4e). The controlled straight cracks, which induce sharply separating crack lips, resulted in a high GF of 2 × 10^6^ within a strain range of 0–10%. Ye et al. [131] developed a strain sensor with unprecedented sensitivity by creating a single crack using a V-notch. This single crack design prevents sensitivity degradation that may occur due to the complex interactions among multiple cracks, resulting in an exceptional sensitivity with a maximum GF of 1.42 × 10^8^. However, the extreme stress concentration results in a narrow working range.

Groove structures provide a wide working range and facilitate easier control over the formation of single cracks. Liao et al. [129] achieved a balance between sensitivity and working range by controlling stress distribution using a PDMS substrate with concave lines and square flat microstructure patterns and employing high-aspect-ratio Ag/NWs as the conductive layer (Figure 4f). Under strain, stress concentration in the concave lines caused plenty of microcracks, and the changes in the concave lines and square flat microstructures lengthened the conductive path for electron transport, resulting in a 136-fold higher GF of 150,000 compared to the un-patterned PDMS substrate. Additionally, the high-aspect-ratio Ag/NWs contributed to a wide working range of 60%.

In addition, with the recent advancement of 3D printing technology, attempts have been made to optimize performance by applying micro-patterns such as notches and grooves to CBSS more easily than with the MEMS process [159]. Shin et al. [130] reported a strain gauge with a sensitivity that was 422% higher than that of a CBSS with a flat surface by directly printing a substrate with groove patterns of various densities using FDM-based 3D printing. Similarly, Chen et al. [52] fabricated CBSS with inverted pyramid microstructure arrays by using template transfer and Au/Ti sputtering after fabricating a microstructure array mold using 3D printing. The fabricated CBSS had a high gauge factor of up to 9327 in the 7.6~10% strain range due to regular linear microcracks induced by stress concentration. Kim et al. [126] reported that by applying inkjet-printed L-shaped micro notch (LMN) to CNT films, which are challenging to control cracks due to their high ductility, they achieved pre-designed full cracking control. The CNT film strain sensors with controlled cracking exhibited an enhanced sensitivity (GF ≈ 73.5) and high linearity (R^2^ > 0.99) over a strain range of 0.01–100%. These stress concentration structures normally limit the working range of CBSS.

Liu et al. [127] developed a 3D CBSS by depositing an Ecoflex-graphite composite conductive layer onto pre-cracked Ecoflex layers fabricated via UVO_3_ exposure. The prefabricated cracks formed straight cracks in the top layer and redistributed stress to regulate the evolution of network cracks in the side layer, achieving high sensitivity (GF = 1084.16), a wide working range (up to 100%) (Figure 4h). This study suggests that applying three-dimensional structures, rather than two-dimensional ones, to CBSS can optimize their performance.

### 4.2. Wrinkle Structure

**Figure 5:**
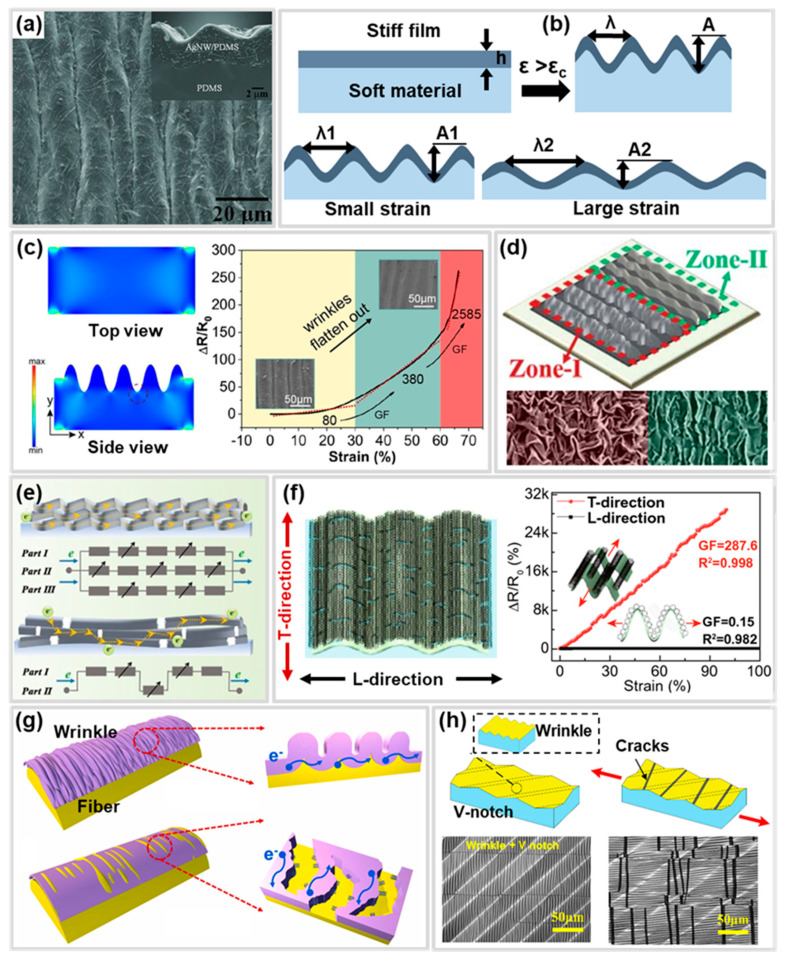
(**a**) SEM images of wrinkle structure [160]; (**b**) Illustration of wrinkle formation with wavelength and amplitude based on pre-strain (**top**), and changes in wavelength and amplitude when small and large strain are applied to wrinkles structure (**bottom**); (**c**) Comparison of stress distribution between plane and wrinkle surface (**left**), wrinkle structure and resistance change according to strain (**right**) [137]; (**d**) Schematic of gradient wrinkle strain sensor that have Zone-l (formed by large strain) and Zone-ll (formed by low strain) [132]; (**e**) Schematic of CBSS with hierarchical wrinkle structure [138]; (**f**) Multidirectional strain sensor using wrinkle structure [134]; (**g**) Fiber sensor that applied wrinkle structure [152]; (**h**) Hierarchical structure strain sensor that integrated notch and wrinkle structure [155].

The wrinkle structure is formed through buckling that occurs during the expansion and shrinkage processes in a bilayer system with different mechanical and geometrical properties (Figure 5a,b) [160,161]. The amplitude and wavelength of the wrinkle structure change with applied strain (Figure 5b), and when the amplitude reaches zero, the rigid layer begins to fracture. Therefore, the wrinkle structure can serve as a functional structure in CBSS, improving the working range by delaying crack generation or propagation in the conductive layer [162].

Zou et al. [137] reported that the use of wave-inspired wrinkle structures can improve the working range of CBSS by forming predictable cracks. The sensor was fabricated by forming a wrinkle structure through a mismatch in the Young’s modulus of PDMS, a silicate layer, and a graphene oxide (GO) layer, followed by gold deposition. Tensile load simulation results showed that stress concentration occurs in the troughs of the wrinkle structure, leading to the generation and propagation of controlled, parallel and unconnected cracks (Figure 5c). Additionally, as the strain increased and the wrinkle structure gradually flattened, cracks progressively expanded from the troughs on both sides toward the center. This contributes to the wide working range of CBSS.

The CBSS must possess both a wide working range and high sensitivity to be applicable to various applications. However, wrinkle structures are limited by their relatively low sensitivity. Chu et al. [132] reported that applying two wrinkle gradient regions with different pre-strains to a CBSS simultaneously improved both the working range and sensitivity (Figure 5d). Zone-I, fabricated with a larger pre-strain, suppressed crack generation and propagation, thereby enhancing the working range. In contrast, Zone-II, fabricated with a smaller pre-strain, enabled crack formation and propagation at lower strain levels, improving sensitivity. Consequently, the sensor achieved ultra-high sensitivity (maximum GF = 167,666.6) and an extended working range (up to 300%). Similarly, Guo et al. [138] fabricated PDMS/MXene/rGO gradient wrinkle strain sensors with different gradient wrinkles by controlling the thickness within the MXene/rGO film, and achieved high sensitivity (GF = 136,124.40) and a wide working range (300%).

To fabricate sensors that are suitable for specific applications, the wrinkle structure employs several strategies, including anisotropic sensing and yarn-shaped substrate. Anisotropic sensing is highly desired for detecting complex multidimensional strains commonly encountered in practical applications. Zhang et al. [134] combined wrinkled and micro-cracked CNT–GO hybrid film with aligned CNT film to fabricate multidirectional strain sensors, achieving high selectivity (6.3) (Figure 5f). To form wrinkles in the CNT–GO hybrid film, CNTs aligned perpendicular to the direction of pre-strain application (L direction) and parallel to the transverse direction (T direction) connected microcracks generated in the CNT–GO hybrid film under tensile strain in the T direction, contributing to high sensitivity (GF = 287.6) and a wide working range (100%). In contrast, the wrinkles formed in the L direction dissipated strain energy by flattening the wrinkles without altering the conductive network of a sensor under strain in the L direction, resulting in low sensitivity (GF = 0.15) in that direction. This contributed to the high selectivity (6.3) of the sensor. These exceptional sensing capabilities of the multidirectional sensor would find potential applications in wearable motion capture devices for emerging wearable electronics and artificial skins. Also, the wrinkle structure can be easily applied to yarns and fibers, which can be easily incorporated into gloves, bands, clothing, etc., and applied to various applications. Sun et al. [32] reported a wrinkle-assisted crack microstructure yarn strain sensor by introducing a wrinkle structure and cracks into a yarn strain sensor through pre-strain, thereby enhancing the working range and sensitivity. Similarly, Zhao et al. [152] reported a fiber-shaped strain sensor that integrates hollow-porous structures with wrinkles and cracks on its surface (Figure 5g). The TPU/fluorescent agent (FA) with hollow-porous structures was fabricated via wet-spinning, followed by a pre-stretching process and CNT deposition to form the wrinkle structure on the surface. This design achieves a wide working range (up to 530% strain), high sensitivity (GF = 57.2), low density (1.017 g·cm⁻^3^), and a low detection limit (0.5% strain). In addition, Zhang et al. [163] reported that the wrinkle structure can be utilized not only to improve the working range but also to enhance the stability. They fabricated CBSS with wrinkles and cracks by depositing Au on an acrylic acid film with a high Poisson’s ratio and then pre-stretching it. During the pre-stretching process, cracks were formed in the direction perpendicular to the wrinkles due to the high Poisson’s ratio of the substrate. During the tensile test process, the crack width increased, but because of Poisson’s ratio, the wrinkles were compressed, and the expansion of the crack tips was suppressed, which ultimately contributed to the improvement of the stability and working range.

Recently, research has been reported on maximizing sensor performance through hierarchical structures that integrate over two functional structures. Ji et al. [155] reported that the integration of a wrinkle structure and a stress concentration structure in a CBSS improved both sensitivity and working range simultaneously. The CBSS, featuring the two integrated structures, was fabricated by pre-stretching PDMS with a 45-degree V-groove array, followed by Au deposition. The cracks induced by stress concentration in the V-groove during tensile process of the sensor contributed to its high sensitivity (GF = 2557.71), while strain absorption through the expansion of wrinkles and crack propagation suppression due to the oblique angle of the V-groove enabled a wide working range (45%) (Figure 5h). In addition, the same researchers fabricated a CBSS with different sensitivities depending on the direction by biaxially pre-stretching a V-groove substrate [164]. The fabricated sensor showed higher selectivity (82.32) and sensitivity (GF_MAX_ is 20,727.46) than the multidirectional sensor that uses a wrinkle structure created through uniaxial stretching [134], due to the structure that combined the V-groove and the anisotropic wrinkle. These hierarchical structure sensors efficiently maximize the performance of CBSS, suggesting their applicability in a variety of practical applications.

### 4.3. Overlap Structure

**Figure 6:**
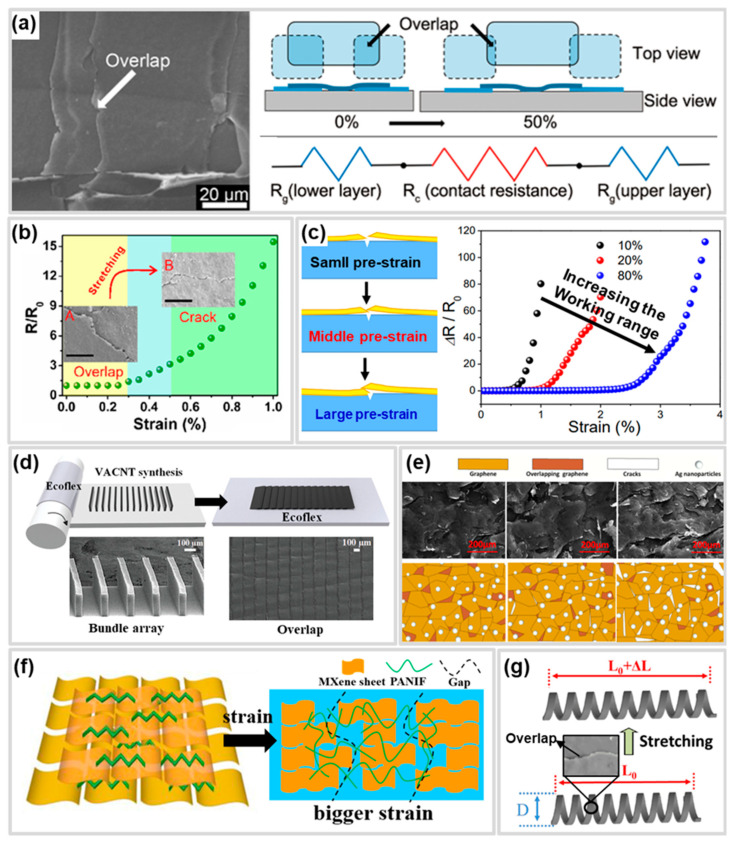
(**a**) SEM image and schematic of overlap structure mechanism [146]; (**b**) Electronical response and SEM image of surface structure change according to applied strain [145]; (**c**) Schematic of overlap structure and sensor performance under difference pre-strain level [94]; (**d**) Overlap structure fabrication method using rolling process [141]; (**e**) SEM image and mechanisms schematic of AgNPs/rGO crack-based strain sensor [147]; (**f**) Schematic of MXene/PANIF nanocomposite strain sensor [139]; (**g**) 3D structure strain sensor combined to overlap structure [143].

The overlap structure consists of conductive layers that have been fractured or deformed through tension and pressure and are overlapped with each other. When applying strain, the overlap structure delays crack generation by decreasing the contact area of the conductive layer (Figure 6a) [146]. Therefore, the overlap structure improves the working range of CBSS by delaying the disconnecting of the conductive path in the conductive layer. The overlap structure has traditionally been regarded as a defect in CBSS, as it impairs sensitivity and linearity. To address this issue, Jung et al. [165] simultaneously applied pre-strain and sensor-extending processes to CBSS, successfully preventing the formation of overlap structures and thereby enhancing sensitivity (GF > 5000) and linearity (R^2^ > 0.99).

However, the overlap structure can be utilized as a strategy to improve the working range of CBSS, as the conductive paths are maintained with a gradual increase in resistance until the separation of the overlapped conductive layers occurs (Figure 6b) [145]. CBSS with overlap structures are manufactured using various processes such as pre-strain and pressing. According to Yang et al. [94] the overlapped area of the conductive layer can be adjusted based on the pre-strain level (Figure 6c). As the pre-strain level increases, the fractured conductive layer caused by cracks exhibits an increase in the overlapped area. Therefore, higher pre-strain levels resulted in an improvement in the working range at CBSS.

Overlap structures can also be fabricated by laminating and pressing conductive layers. Meng et al. [142] reported a strain sensor with a maximum working range of 25% and GF 150 through layer-by-layer laminating of graphene platelets (GnP) film onto polyurethane (PU) film. Also, Lee et al. [141] enhanced the working range and sensitivity by forming an overlap structured CNT sheet on Ecoflex using vertically aligned CNT bundles through a rolling process. This sensor achieved a wide working range (>145%) and high sensitivity (GF = 42,300 at 125–145%) as the overlapped regions decreased due to sliding between CNT bundles under strain, followed by separation (Figure 6d). Similarly, the same group improved the working range up to 500% by arranging a zig-zag CNT bundle array and forming an overlap structure with adjacent bundles [144].

The overlap structure is inherently limited by low sensitivity due to its mechanism. The laminating of nanoparticles into an overlapping structure is a method to improve sensitivity in overlap structure. Yang et al. [147] fabricated a nanoparticle-embedded reduced graphene oxide (rGO) by drop casting a mixture of AgNP and graphene oxide (GO) onto PDMS and reducing GO through laser scribing. AgNPs connect the surface and inside of randomly overlapped graphene flakes with conductive bridges to decrease the initial resistance and change the layer resistance (Figure 6e) [147]. Because of this, the AgNP/rGO/PDMS strain sensor has shown an improved working range and sensitivity compared to rGO/PET [166] and rGO/PDMS [167] strain sensors fabricated by laser scribing.

A study has been reported to simultaneously achieve performance sensitivity and working range of CBSS through an overlap structure for various applications. Chao et al. [139] fabricated a MXene/polyaniline fiber (MXene/PANIF) nanocomposite sensor by applying pre-strain to MXene and coating it on PANIF. The MXene sheets were partially overlapped, and the PANIF connected MXene sheet (Figure 6f) [139]. Due to the reversible slippage of the MXene/PANIF sheets, the sensor achieved high sensitivity (GF = 2369.1) and a wide working range (up to 80%). Likewise, a 3D-printed helical structure sensor with an overlap design has been reported for applications in wearable healthcare devices (Figure 6g) [143]. The above studies on CBSS optimized with overlap structures have demonstrated their potential to enhance the diversity of applications.

### 4.4. Porous Structure

**Figure 7:**
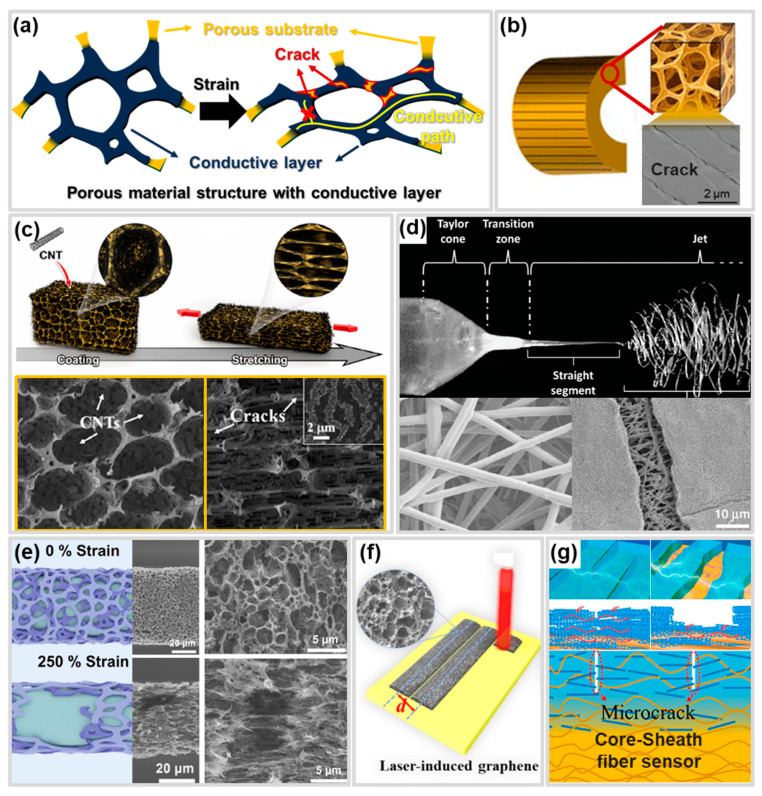
(**a**) Schematic illustration of porous structure strain sensor mechanisms; (**b**) Image of Au/PU sponge strain sensor [171]; (**c**) SEM image and schematic illustration of TPU/CNT sensor in tensile strain [64]; (**d**) Mechanisms of electrospinning process and SEM image of CBSS with electrospun fibrous membrane [176,179]; (**e**) Schematic and SEM images showing the morphological evolution of the CNT/SEBS/PU fiber strain sensor as a function of tensile strain [185]; (**f**) Fabrication method of Laser-induced graphene to porous conductive layer [188]; (**g**) Schematic of crack-based core-sheath fiber strain sensor mechanisms [151].

Porous structure is defined as a structure in which pores are formed on the surface or inside the material. This structure can be applied to CBSS as a functional structure that can improve the working range by delaying the complete destruction of the conductive network due to crack generation (Figure 7a). Materials with porous structures have higher fracture strains than continuous materials due to their lower mass density and Young’s modulus, 3D rotation that occurs during deformation, etc. In addition, the porous structure weakens the destructive effect of the conductive network by forming a new conductive network through the evolution of pores under tension and compression and contributes to and maintains the conductive path through non-destroyed pore walls in the conductive network [64,168,169,170,171,172,173]. Wu et al. [171] demonstrated that conductive network and pathway of a Au/PU Porous CBSS change before and after compressive strain is applied (Figure 7b). This sensor is fabricated using commercial PU(Polyurethane) sponge [172]. CBSS with porous structure are fabricated using flexible materials, including sponges and fibrous membranes [173,174,175]. Zhu et al. [64] reported TPU 3D porous skeleton structure/CNT multimodal sensor to improve the working range of CBSS (Figure 7c). The fabricated porous skeleton structure is characterized by the presence of tiny microscale pores within the porous skeleton, which evolve under tensile strain. The excellent mechanical properties of this porous structure and the maintenance of the conduction path through the evolution of the pores contribute to the performance of the working range of 900% strain, while microcracks that generated in the CNTs induce a larger resistance increase under tiny deformation, showing an ultra-low detection limit of 0.01%. Fibrous membrane, unlike sponges, allows precise control over their 3D porous network. The electrospinning process (Figure 7d) is an eco-friendly, simple and efficient method for fabricating various types of fibrous membranes [176,177]. Additionally, the process enables the production of fibrous membranes with excellent quality, high porosity, and uniformity, which increase the specific surface area, significantly improving the performance of CBSS [169,178]. Sun et al. [179] deposited CNTs and AgNWs on electrospun porous TPU fiber mats via vacuum-assisted filtration to achieve both high sensitivity and wide working range. Pre-stretching treatment at 135–171% strain constructed a microcrack structure, providing the sensor with combined characteristics of an ultrahigh sensitivity (>11 × 10^4^ within 135–171% strain) and wide working range (0–171% strain). Furthermore, they achieved a fast response time (~65 ms), small hysteresis, and superior repeatability (>2000 cycles). CBSS with porous structure can be fabricated in various forms using flexible materials, including foam [180,181,182,183], and film [184]. Applying a porous structure in the conductive layer, unlike substrate layer, can improve working range by reducing the Young’s modulus difference between the conductive layer and the substrate and by improving the mechanical properties of the conductive layer. Gu et al. [185] fabricated a CNT/Styrene-ethylene-butylene-styrene (SEBS)/PU fiber strain sensor by depositing porous CNT/SEBS conductive film on the PU fiber surface using breath figure method combined with spray method (Figure 7e). The 3D conductive network of the porous structure of the conductive layer can detect deformations until serious damage is caused by the growth of cracks. Therefore, fibrous strain sensors that have porous structure can have a working range up to 240% strain, which is wider than non-porous fibrous strain sensors. Porous conductive layer can be easily fabricated using LIG process (Figure 7f) which produces a 3D-porous structure [186,187].

CBSS with porous structures composed of conductive layers and substrates may have limited working ranges due to poor interface compatibility. Qu et al. [151] solved this problem by fabricating a crack-based core-sheath fiber strain sensor using coaxial wet spinning on TPU and CNTs added TPU (Figure 7g). During the fabrication process, the interfacial conductive network was formed due to the interpenetration between the porous TPU and the CNTs/TPU. The interfacial conductive network contributed to transport electrons even if the CNTs/TPU bridge in the CNTs/TPU network is destroyed. Therefore, the crack-based core-sheath fiber strain sensor reached a working range of 500%, GF of 66,600, and repeatability over 5000 tensile cycles.

These porous structures can be formed together with other functional structures to further enhance performance. For example, CBSS with improved working range by simultaneously applying a wrinkle structure and the porous structure have been reported. For example, Zhao et al. [170] developed a CBSS with a wrinkle structure by fabricating a porous TPU fiber with an improved breaking strain (805%) through a wet spinning process and then coating MXene and MWCNT through two pre-stretching processes. The CBSS fabricated in this way exhibited a high sensitivity (GF ≈ 277) and a wide working range (510%) due to the good stretchability of the porous TPU fiber and the effects of the multidimensional conductive material and the wrinkle structure. The above studies suggest that porous structures can be applied to the substrate or conductive layer of CBSS to improve the working range through the improvement of mechanical properties or the formation of effective conductive paths and new conductive paths.

### 4.5. Performance Control Through Numerical Analysis of Functional Structure

**Figure 8:**
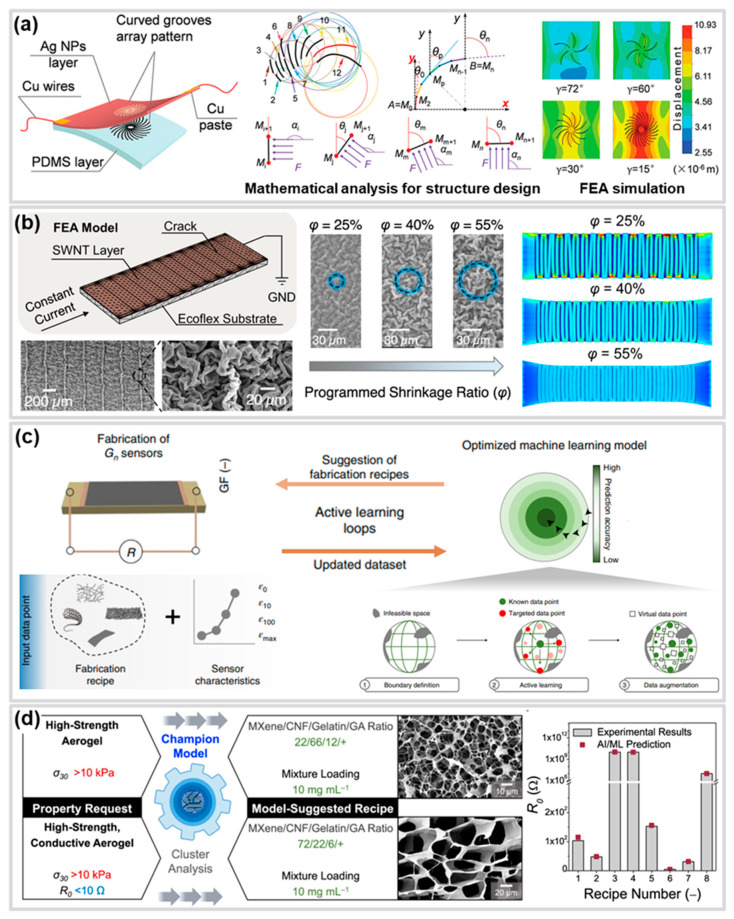
(**a**) Optimal design of a scorpion leg slit inspired structures (1 to 12) using mathematical theory and FEA simulation for CBSS [128]; (**b**) Computation-guided sensor designs using programmed crack array within micro-crumples (PCAM) [69]; (**c**) CBSS recipe design process with performance suitable for soft machines using machine learning [70]; (**d**) Automatic design process of aerogel recipes with performance suited to specific applications using machine learning [189].

Numerical models and machine learning models applicable to the functional structures of CBSS play a crucial role in analyzing sensor mechanisms and optimizing performance. The various shapes and configurations of functional structures have a complex influence on several performance metrics, such as the sensitivity and working range of the sensors, making precise analysis essential for optimizing them to achieve desired performance. For these reasons, numerical models and machine learning models serve as tools to effectively identify correlations that are difficult to uncover through experiments and theoretical analysis alone. These models contribute to deriving optimized structural designs or predicting the sensitivity and working range of sensors in advance. In previous studies, researchers primarily utilized numerical simulations to explain the mechanisms by which functional structures influence the performance of CBSS [125,131,137]. However, recent research has focused on integrating mathematical and physical theories with simulations to optimize desired performance or propose prediction models, aiming to enhance the research efficiency in terms of time and cost. Liu et al. [128] conducted mathematical analysis using calculus theory to design a sensor structure that mimics the scorpion slit, capable of maximum deformation under vibrations applied from all directions. Additionally, finite element analysis (FEA) was used to investigate stress distribution based on design parameters, optimizing the slit shape, number, and angle. This optimization led to the fabrication of a high-sensitivity sensor with a GF of 18,000 at 0.46–0.65% strain (Figure 8a). Furthermore, Yang et al. [69] examined how controllable crack density and micro-crumple parameters affect sensor performance. These parameters could be computationally determined before sensor fabrication, enabling the realization of computation-guided sensor designs. This design approach involved inputting computational parameters into FEA-based physical modeling, allowing the prediction of sensing performance without experimental trials. The predicted results were reported to be in strong agreement with actual experimental data (Figure 8b). Additionally, Yang et al. [70] fabricated CBSS using Mxene nanosheets, SWCNT, and PVA binders, collecting performance data based on fabrication recipes. They developed and trained a machine learning model using a three-stage framework comprising support vector machine (SVM), active learning, and data augmentation. The model demonstrated the ability to propose fabrication recipes for sensors with specific sensitivity and working range requirements, based on design inputs. This approach highlights the potential to significantly reduce trial-and-error efforts and improve efficiency in the strain sensor design process (Figure 8c). Such machine learning-based performance predictions are not limited to CBSS but have also demonstrated significant potential in proposing recipes to meet specific performance requirements such as high electrical conductivity, strength, and pressure insensitivity—for designing suitable aerogels in the field of wearable thermal management (Figure 8d) [189]. In conclusion, this discussion highlights the essential role of numerical simulations and machine learning in optimizing CBSS, emphasizing their ability to significantly enhance research efficiency and drive innovation in sensor design.

## 5. Application of Crack-Based Strain Sensors

Performance optimization through various CBSS strategies enables accurate monitoring of physiological signals and motions, including pulse [101,190], breathing [39], facial muscle movement [39], cerebral blood flow [49], vibrations from humans and machinery [128,191,192,193], which require high sensitivity at small strain, and joint movements in humans or robots, which require a wide working range [59,69,194]. These characteristics make CBSS promising for applications in physiology and motion monitoring [39,49,101,190,192,194,195], human–machine interfaces [196,197,198], and structural health monitoring [41,128,192,193,199]. In this section, we present applications of CBSS in these fields to demonstrate their potential across various domains.

### 5.1. Physiology and Motion Monitoring

The high sensitivity resulting from cracks in CBSS, combined with flexible and stretchable component materials, makes them advantageous for direct skin attachment and precise detection of small deformations, such as physiological signals [39,49,101,190,192,194,195].

**Figure 9:**
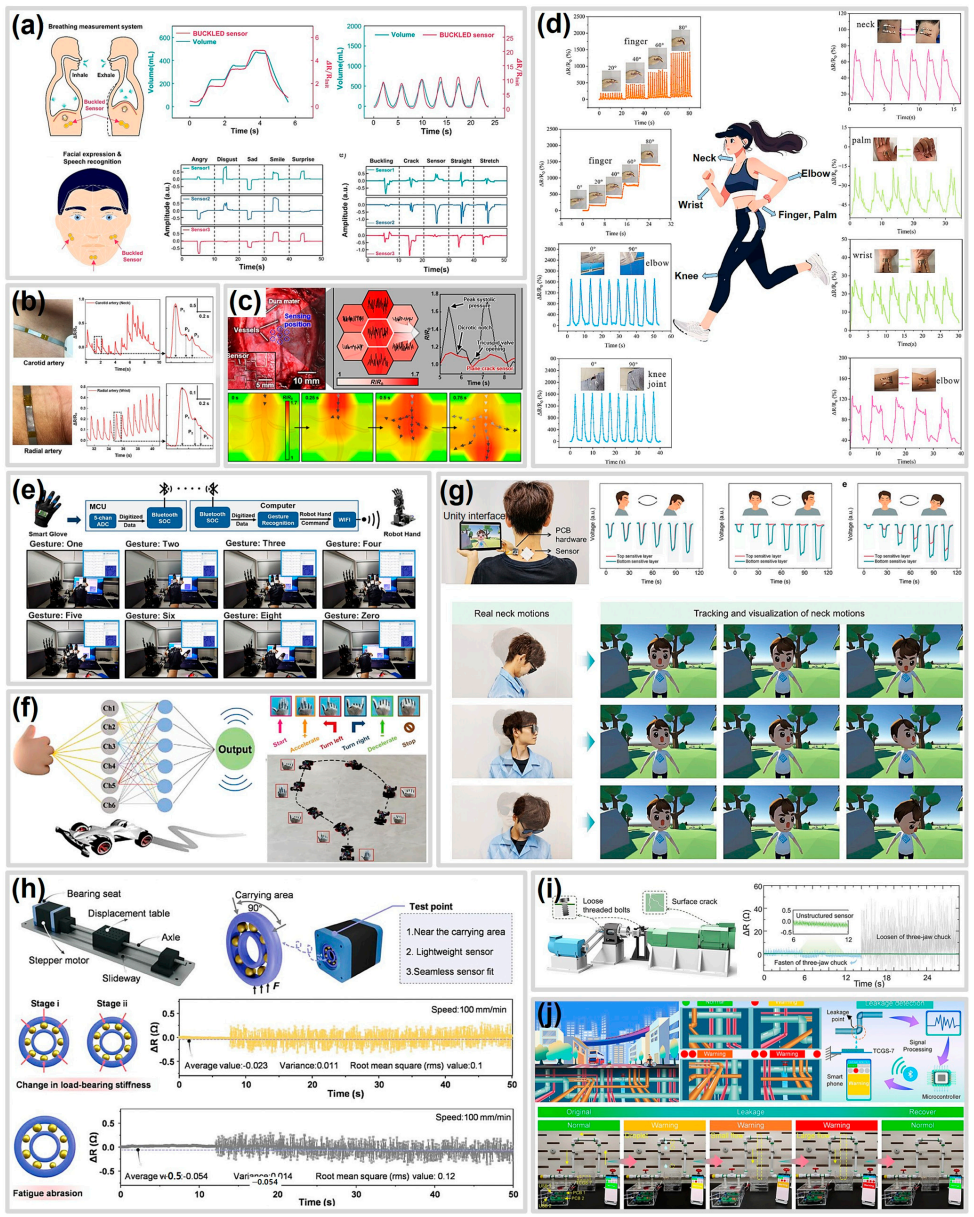
(**a**) Breathing volume and facial strain monitoring [39]. (**b**) Arterial pulse monitoring [101]. (**c**) Cerebrovascular status monitoring [49]. (**d**) Human motion monitoring [194]. (**e**) Robot hand control using hand gestures [196]. (**f**) Car control using hand gestures [197]. (**g**) 3D motion capture for neck motion monitoring [198]. (**h**) Rolling bearing condition monitoring [192]. (**i**) Machine tool condition monitoring [193]. (**j**) Pipeline leakage monitoring [199].

Figure 9a shows breathing volume and facial strain signals measured by CBSS [39]. CBSS attached to the abdomen indirectly measured breathing volume based on abdominal skin strain. Sensor resistance increased proportionally to breathing volume, showing a high correlation of 0.94 and approximately 90% statistical accuracy. Additionally, sensors attached to the chin and cheeks measured facial strain during expressions and pronunciation. Data were processed using a 1D convolutional neural network, accurately distinguishing facial expressions (accuracy is 0.97) and pronunciation (accuracy is 0.95).

CBSS can also monitor arterial pulse waveforms [101,190]. Figure 9b illustrates pulse waveform monitoring of the radial and carotid arteries with CBSS attached to the wrist and neck [101]. The sensor accurately differentiated arterial pulse components (P1 is Incident, P2 is Tidal, P3 is Diastolic), enabling calculation of indices (radial augmentation index, diastolic augmentation index, digital volume pulse time) critical for assessing cardiovascular health. Similarly, CBSS directly attached to the brain provided real-time monitoring of cerebrovascular pulse pressure, rate, and blood flow, requiring extremely small strain detection limits (Figure 9c) [49]. These results highlight CBSS’s potential for real-time monitoring of vascular health related to vasoocclusion and intracranial pressure from traumatic brain injury.

Moreover, CBSS optimized for improved working range can effectively monitor human motion. Figure 9d presents motion monitoring results from CBSS attached to fingers, elbows, knees, neck, palms, and wrists [194]. CBSS demonstrated sensitivity to subtle movements (neck, palm, wrist) and adequately measured resistance changes from large joint movements (finger, elbow, knee). These examples illustrate the applicability of CBSS in physiology and motion monitoring.

### 5.2. Human–Machine Interface

The exceptional motion monitoring capability of CBSS facilitates converting human movements into electrical signals, enabling machine control and positioning CBSS as highly suitable for Human–Machine Interface (HMI) applications. Figure 9e depicts robotic hand control through a smart glove integrated with CBSS [196]. The smart glove sensors capture finger gestures, converting them into digital signals via an MCU module. Signals are then wirelessly transmitted to control the robotic hand using a 1D-CNN model. Similarly, CBSS-integrated smart gloves enable gesture-based car control (Figure 9f) [197].

CBSS also supports visualizing human motion in virtual environments. Figure 9g illustrates real-time motion capture using CBSS. Xu et al. [198] developed a CBSS capable of simultaneously detecting stretching amplitude and direction by stacking elastomer meshes with varying fiber orientations. Distinct electrical signals were generated according to fiber orientation, enabling a 3D motion capture system to visualize and track neck movements. These applications underscore CBSS’s significant potential for HMI.

### 5.3. Structural Health Monitoring

CBSS’s superior vibration detection, attributed to high sensitivity, offers great potential for structural health monitoring of machinery and infrastructure. Li et al. [192] applied ultra-sensitive CBSS (sensitivity > 10^5^, detection limit is 8.3 µm) to monitor rolling bearing conditions (Figure 9h). Under normal loads, bearing vibration waveforms exhibited consistent regularity, whereas worn bearings displayed increased randomness in vibration patterns.

Similarly, Wang et al. [193] implemented CBSS with high sensitivity (GF is 657.36) and broad frequency response (103 Hz) in machine tools. Sensor output resistance distinctly varied during normal operation versus bolt loosening or sudden component shedding impacts (Figure 9i). These findings emphasize CBSS’s potential for machine health monitoring, including wear detection, loosened components, part shedding, and surface crack identification.

Furthermore, CBSS is advantageous for infrastructure monitoring applications like buildings, bridges, and pipelines [41,128,199]. Liu et al. [128] developed omnidirectional CBSS inspired by the scorpion’s curved slit, effectively detecting small strains from loose screws, corrosion, and random cracks critical for structural integrity. Zhou et al. [199] applied CBSS for liquid leakage detection in pipelines (Figure 9j), combining scorpion slit structures with microporous patterns. The resulting sensor, with a sensitivity of 218.13 at 2% strain and superhydrophobic properties, provided stable resistance signals for leak detection, enabling smartphone-based leak alerts. These examples highlight CBSS’s significant advantages for structural health monitoring applications.

## 6. Challenges and Future Perspectives

### 6.1. Challenges in Crack-Based Strain Sensors

Despite the significant advancements in CBSS technology, there remain several challenges that need to be addressed to achieve the full potential of these sensors in practical applications:

#### 6.1.1. Long-Term Stability and Durability

Cracks in CBSS significantly contribute to their high sensitivity. However, these cracks can be influenced by external environmental factors such as humidity [200,201], temperature [202,203,204,205], and airflow [206,207], which can alter crack density, depth, and width. Such alterations lead to unwanted performance degradation [106,208]. Additionally, continuous sensor usage often promotes crack growth and propagation, potentially damaging the conductive layer or substrate and raising concerns regarding sensor stability and durability.

Previous studies have sought to address these issues by enhancing sensor durability and stability through methods such as sensor packaging [209], introduction of crack-suppression layers [109], and surface structure modifications [210,211,212]. Efforts have also been made to eliminate noise via compensation circuits [213] and signal processing techniques [214]. However, these approaches frequently result in reduced sensitivity [109,209], and increased sensor system size and complexity. Therefore, to expand CBSS applications across various fields, it is essential to develop strategies that improve durability and long-term stability without compromising sensor performance.

#### 6.1.2. Fabrication Process and Scalability

CBSS utilizes a unique sensing mechanism based on micro- to nanoscale cracks. Precisely controlling these cracks is crucial for ensuring the reproducibility and reliability of the sensors. However, current technologies for precise crack fabrication often rely on complex and expensive MEMS processes such as photolithography and etching [130,215]. These intricate methods are unsuitable for mass production and face scalability limitations. Alternative techniques, such as roll-to-roll printing [216], laser scribing [217], and additive manufacturing [130] have demonstrated potential for scalability in mass production but suffer from issues such as lower precision and material wastage [218,219]. Therefore, developing cost-effective and scalable fabrication processes capable of precise crack control is imperative to enable the commercialization of CBSS.

#### 6.1.3. Integration with Electronic Devices

To commercialize CBSS as functional devices, integration with components such as batteries, wireless communication modules, and data processors is essential. However, mechanical property mismatches between the sensor and electronic component pose significant challenges in achieving seamless integration [220]. Flexible printed circuit boards (fPCBs) have been employed to address some of these challenges, facilitating integration with CBSS [221,222]. Nonetheless, the rigid characteristic of the modules within fPCBs remains an issue, necessitating further innovations to achieve effective integration without compromising device performance.

### 6.2. Future Perspectives

The rapid advancement of CBSS has enabled its application in various fields, including physiology and motion monitoring, human–machine interfaces, and structural health monitoring. However, despite significant progress, several challenges remain. Existing CBSS crack types, particularly those based on straight and network cracks, often face limitations due to the trade-off relationship between sensitivity and working range. Moreover, optimizing this trade-off continues to be a critical challenge in practical applications, requiring considerable time and effort from researchers. To overcome these issues, researchers are exploring new approaches such as advanced cracks, hierarchical cracks, hierarchical structures, and optimization using numerical analysis and data-driven methods. This section discusses promising future directions expected to drive the next generation of CBSS technology.

#### 6.2.1. Advanced Cracks

Advanced crack architectures address the limitations of conventional straight or network cracks by offering higher strain resolution and improved repeatability. Traditional CBSS designs, while highly sensitive, can struggle with micro-scale deformations or maintain consistent crack closure over multiple cycles. By introducing new crack geometries and specialized substrates, researchers aim to achieve both high gauge factors and minimal hysteresis, thereby enabling precise detection of subtle strains in fields like wearable health monitoring and soft robotics.

A notable example is the meta crack, which employs a negative Poisson’s ratio (auxetic) substrate to induce a unique crack-opening mechanism [49]. Unlike standard systems where Poisson effect can partially reconnect crack edges under tension, auxetic substrates expand laterally when stretched, widening the crack gap and preventing edge contact. This results in exceptionally high sensitivity even at strain levels of 10^−5^ and large resistance changes under minimal deformation. Ongoing studies of auxetic materials, hybrid film structures, and other “advanced cracks” are expected to propel CBSS technology toward ultra-sensitive and robust performance across diverse applications.

#### 6.2.2. Hierarchical Cracks

A hierarchical crack is a crack type that forms in the conductive layer, where different geometrical factors or crack types occur simultaneously [24,80,223]. Hierarchical cracks can optimize sensor performance by combining multiple geometrical factors and crack types within a single conductive layer. For example, Cheng et al. [24] reported Au/graphene composite films (AGCFs) featuring hierarchical cracks. During the tensile process, the fabricated AGCF sensor initially formed primary cracks, followed by the growth of branch-like microcracks around them, ultimately resulting in a fractal-like crack pattern. This branch-like fractal structure contributed to the sensor’s high sensitivity and low detection limit by allowing numerous microcracks to respond sensitively even to small deformations. As another example, Wang et al. [80] induced the simultaneous formation of deep straight cracks and shallow network cracks in an MXene/AgNW layer by controlling the UV-ozone treatment time for PDMS. Cracks of different types and depths contributed, respectively, to improved sensitivity and an expanded working range, enabling the fabrication of a CBSS with a high gauge factor (GF ≈ 244) over a wide linear strain range (ε = 60%, R^2^ ≈ 99.25%). These examples demonstrate the potential of hierarchical cracks as an innovative approach to optimize the performance of CBSS.

#### 6.2.3. Hierarchical Structures

Functional structures are important in enhancing the performance (sensitivity and working range) of CBSS. Stress concentration structures are utilized to improve sensitivity [125], while wrinkles, overlaps, and porous structures are effective in expanding the working range [64,137,144]. However, to meet the performance requirements of various applications, it is essential to achieve a balanced optimization of the trade-off between sensitivity and working range. To achieve this, hierarchical structures combining two or more functional structures have emerged as a promising solution. These hierarchical structures integrate the strengths of each functional structure to achieve sensitivity and working range tailored to specific requirements [152,155]. Realizing this potential necessitates further research into the design and optimization of hierarchical structures, which will accelerate the development of next-generation CBSS.

#### 6.2.4. Optimization Using Numerical Analysis and Data-Driven Methods

The performance of CBSS is influenced by the constituent materials, crack type, geometric factors, and functional structures. Numerical simulations and a thorough understanding of the mathematical and physical theories related to these variables help improve and optimize CBSS performance through the design of programmable cracks [69,116]. However, the complex interactions between crack variables pose significant challenges in predicting and enhancing sensor performance. Therefore, data-driven prediction methods, such as machine learning [70] and knowledge graphs [72] offer promising solutions for more efficient performance optimization. By adopting these approaches, it is anticipated that the time and cost required for optimizing CBSS performance can be significantly reduced, ultimately providing practical value across various application fields.

## 7. Conclusions

In summary, CBSS are redefining how mechanical cracks can be harnessed as functional elements to achieve ultra-high sensitivity and extensive working ranges. This review has dissected the fundamental mechanisms of different crack types—straight, network, kirigami, and meta—and their interplay with geometrical factors such as asperity, depth, and density. We also explored how functional structures including stress concentration features, wrinkles, overlaps, and porous architectures can be leveraged to balance trade-offs between sensitivity and working range.

Furthermore, recent progress in computational modeling and machine learning provides promising avenues for cost-effective, time-efficient sensor design and optimization. Continued research in these areas, combined with innovations in scalable manufacturing and integration with flexible electronics, will propel CBSS technologies toward wider adoption across numerous sectors from wearable health monitoring and soft robotics to structural health assessment. By embracing an integrated approach that includes both experimental insight and computationally guided strategies, the next generation of CBSS shows potential to deliver transformative capabilities in diverse real-world applications.

## Data Availability

Not applicable.

## Abbreviations

The following abbreviations are used in this manuscript:

a-C	Amorphous carbon
AgNW	Silver nanowires
Al_2_O_3_	Aluminum oxide
Au	Gold
CBSS	Crack-based strain sensors
CNT	Carbon nanotube
Cr	Chromium
DS	Dragon skin
FA	Fluorescent agent
FEA	Finite element analysis
FE	Finite element
fPCB	Flexible printed circuit board
FSR	Fluorosilicone rubber
GF	Gauge factor
GnP	Graphene platelets
GO	Graphene oxide
LIG	Laser-induced graphene
LTP	Laser transmission pyrolysis
MEMS-	Microelectromechanical systems
Mo	Molybdenum
MoO_3_	Molybdenum trioxide
MXene	New intriguing family of 2D transition metal carbides, nitrides, and carbonitrides
O_3_	Ozone
PANIF	Polyaniline fiber
PBAT	Polybutylene adipate terephthalate
PCAM	Programmed crack array within micro-crumples
PDMS	Polydimethylsiloxane
PDCA	Probability distribution based cellular automata method
PEDOT:PSS	Poly(3,4-ethylenedioxythiophene) polystyrene sulfonate
PLA	Polylactic acid
Pt	Platinum
PU	Polyurethane
PUA	Polyurethane acrylate
rGO	Reduced graphene oxide
SEBS	Styrene-ethylene-butylene-styrene
SiO_x_	Silicon oxide
SVM	Support vector machine
SWCNT	Single-walled carbon nanotube
UV	Ultraviolet
HMI	Human–machine interface
